# Improved renal recovery in patients with atypical hemolytic uremic syndrome following rapid initiation of eculizumab treatment

**DOI:** 10.1007/s40620-016-0288-3

**Published:** 2016-03-19

**Authors:** Johan Vande Walle, Yahsou Delmas, Gianluigi Ardissino, Jimmy Wang, John F. Kincaid, Herman Haller

**Affiliations:** 1 University Hospital Ghent, De Pintelaan 185, 9000 Ghent, Belgium; 2 Centre Hospitalier Universitaire de Bordeaux, Bordeaux, France; 3 Ospedale Maggiore Policlinico, Milan, Italy; 4 Alexion Pharmaceuticals, Cheshire, CT USA; 5 Medical School Hannover, Hannover, Germany

**Keywords:** Atypical hemolytic uremic syndrome, Chronic kidney disease, Eculizumab, Thrombocytopenia, Thrombotic microangiopathy

## Abstract

**Background:**

Eculizumab is approved for atypical hemolytic uremic syndrome (aHUS). Guidelines discuss the importance of prompt treatment. We report a post hoc analysis investigating the effect of baseline factors, including patient characteristics and time from the latest aHUS manifestation to eculizumab initiation, on change from baseline in estimated glomerular filtration rate (eGFR) and other outcomes.

**Methods:**

Data were pooled from four phase 2, open-label, single-arm, prospective clinical studies of eculizumab for patients with aHUS. Multivariate regressions identified predictors of eGFR change from baseline. The proportion of patients achieving sustained eGFR increase (defined: ≥15 ml/min/1.73 m^2^ for ≥28 days) and platelet count normalization were evaluated 1 year post-treatment. Baseline characteristics and eGFR outcomes were summarized by time to treatment from last aHUS manifestation [≤7 days (*n* = 21) versus >7 days (*n* = 76)].

**Results:**

Baseline eGFR were similar between groups. Multivariate regression analysis demonstrated time from aHUS manifestation to eculizumab treatment, age, baseline lactate dehydrogenase (LDH) and baseline hemoglobin were independently predictive of eGFR change from baseline. Mean eGFR change from baseline at 1 year was significantly higher in patients treated in ≤7 days than >7 days (57 vs. 23 ml/min/1.73 m^2^, p = 0.0098). After 1 year, 17/21 and 36/76 patients in the ≤7 and >7 day groups, respectively, achieved a sustained increase in eGFR. Mean time to platelet count normalization was similar between groups.

**Conclusions:**

Younger age, higher baseline LDH and lower baseline hemoglobin were associated with greater eGFR improvements. Early eculizumab initiation led to improved renal recovery, demonstrating the importance of rapid diagnosis and treatment of patients with aHUS.

**Electronic supplementary material:**

The online version of this article (doi:10.1007/s40620-016-0288-3) contains supplementary material, which is available to authorized users.

## Introduction

Atypical hemolytic uremic syndrome (aHUS) is a life-threatening condition [1] caused by uncontrolled activation of the alternative complement pathway that results in damage to endothelial cells and systemic thrombotic microangiopathy (TMA) [2–4]. Approximately 60 % of patients with aHUS have an identified complement regulatory protein dysfunction or mutation in a complement gene [3, 5]. Progression of aHUS can be rapid and severe, and symptoms are characterized by hemolytic anemia, thrombocytopenia and organ damage [3]. The disease predominantly affects the renal vasculature, but ongoing TMA can also impair other organs including the brain, heart, intestines, pancreas, and lungs [2, 3]. Prior to the availability of eculizumab, over 50 % of patients with aHUS died, required dialysis or developed permanent kidney damage within the first year after diagnosis [3].

The rapid progression of TMA, associated with potentially irreversible damage to organs in patients with aHUS, indicates a need for urgent treatment. TMA may lead to acute renal dysfunction in early disease, and evidence suggests acute kidney injury (AKI) and chronic kidney disease (CKD) are closely interrelated, with each being a risk factor for the other [6]. Plasma exchange/plasma infusion (PE/PI) has been used to manage aHUS, but there is no prospective clinical trial evidence showing its efficacy. PE/PI does not resolve the underlying disease, and often fails to prevent progression to end-stage renal disease [1]. Persistence of an inflammatory environment can impair kidney regeneration and further promote the development of CKD [7] in both native and transplanted kidneys [1, 3, 8].

Eculizumab is a recombinant humanized monoclonal antibody that blocks the cleavage of C5, preventing formation of the proinflammatory peptide C5a and the cytotoxic membrane attack complex C5b-9 [2]. Multiple cases and four published prospective clinical studies conducted in patients with aHUS have shown efficacy of eculizumab in aHUS [1, 9, 10], which has led to its approval for the treatment of this disorder. The first trial enrolled patients with progressive TMA (n = 17) and the second enrolled patients receiving long-term PE/PI (n = 20). Both trials met their primary endpoints at 26 weeks: first trial, platelet count normalization, and second trial, TMA event-free status (defined as no decrease in the platelet count of >25 %, no PE/PI, and no initiation of dialysis). At the 1-year data cutoff, the mean increase in platelet count from baseline was 91 × 10^9^/l [95 % confidence interval (CI) 67–116] in the first trial; TMA event–free status for ≥12 weeks was achieved in 85 % of patients in the second trial. Both trials achieved normalization of platelet count and lactate dehydrogenase (LDH) levels (88 and 90 % respectively) [10]. A recent follow-up has shown benefits are maintained at 2 years, with median eculizumab treatment of 100 and 114 weeks in the two trials, respectively [11]. The third trial enrolled 41 adult patients, while the fourth enrolled 22 patients aged 1 month to <18 years. Initial treatment was for 26 weeks with the possibility of continuing eculizumab treatment in a long-term follow-up. In the adult study, 73 % of patients met the primary endpoint, defined as normalization of platelets and LDH and <25 % increase from baseline in serum creatinine levels at 26 weeks [1]. Results from the pediatric study showed 64 % of patients met the primary endpoint, defined as normalization of platelets and LDH and ≥25 % improvement from baseline in serum creatinine levels at 26 weeks [1].

Guidelines recommend children and adults with a clinically definitive aHUS diagnosis be treated with eculizumab ideally within 24 h [9, 12]. It has also been suggested to consider no more than 5 daily plasma exchanges where further investigations are required to confirm aHUS diagnosis. A lack of normalization of platelet count and LDH or improvement in serum creatinine levels within this time is an additional indicator for the switch to eculizumab [9, 12]. Therefore, it is reasonable that patients presenting with TMA as a consequence of aHUS start eculizumab in less than a week.

While the timing of therapy initiation for aHUS has been investigated in small patient cohorts, it remains to be studied in a substantive population [9]. We therefore carried out a post hoc analysis of pooled data from four clinical trials of eculizumab to evaluate the impact of time from onset of last aHUS manifestation to treatment with eculizumab, as well as other factors, on renal and hematological outcomes in patients with aHUS.

## Methods

Data were pooled from the four previously described phase 2, open-label, single-arm, prospective clinical studies which enrolled patients aged 1 month to 80 years [1, 10]. Inclusion required a documented date of onset of symptoms of TMA and a baseline estimated glomerular filtration rate (eGFR) of <90 ml/min/1.73 m^2^. All patients were receiving eculizumab for the first time. In this pooled analysis, three major clinical outcomes were considered: (1) eGFR change from baseline at 1 week and monthly over 1 year, (2) proportion of patients achieving a sustained eGFR increase of ≥15 ml/min/1.73 m^2^ (improvement maintained for at least 28 days) from baseline to 1 year of follow-up, and (3) time to platelet count normalization (≥150 × 10^9^/l). Baseline was defined as: at enrollment prior to first eculizumab dose. It should be noted that when patients were on dialysis, their eGFR was imputed to 10 ml/min/1.73 m^2^.

In order to explore in a clinically relevant and dichotomous way the effect of time from the latest aHUS manifestation to initiation of eculizumab treatment, results were stratified according to whether patients had started eculizumab treatment ≤7 or >7 days after the onset of the latest aHUS manifestation (herein referred to as time to treatment). Baseline characteristics were compared between the ≤7 and >7 day groups using the Wilcoxon rank-sum test for continuous variables and the Fisher exact test for categorical variables. The difference between the ≤7 and >7 day groups in time to platelet count normalization was tested using the two-group *t* test. To test the difference between the two groups (≤7 and >7 day) on the proportion of patients achieving a sustained eGFR increase of ≥15 ml/min/1.73 m^2^, Fisher’s exact test was performed for each visit.

Multivariate regressions using repeated measures analysis were performed to identify predictors of change in eGFR from baseline through 1 year. Baseline eGFR and trial visit (scheduled post-dose visits in months) were always kept in the model as the dependent variable was change from baseline in eGFR and the analysis was a repeated measures analysis over post-dose visits up to 12 months. Ten other baseline variables or patient characteristics were tested, including six categorical variables: age group (<18 vs. ≥18 years), mutation (complement factor H [CFH] mutation, non-CFH mutation, and no mutation), history of TMA manifestation (single vs. multiple), transplant history (yes vs. no), dialysis at baseline (yes vs. no), PE/PI at baseline (yes vs. no), as well as four continuous variables: time to treatment, baseline platelet count, baseline LDH, and baseline hemoglobin. Selection of variables was performed using a forward stepwise procedure to identify the main effects and their interaction terms for inclusion in the final model. In order to test the effect of time to treatment as both a continuous and a categorical variable, after the final model was identified (including time to treatment), it was repeated replacing continuous time to treatment with categorical time to treatment (≤7 vs. >7 days).

The raw mean changes in eGFR from baseline over time up to 12 months are presented for the two dichotomized time to treatment groups (≤7 and >7 days). To evaluate the differences in eGFR change from baseline between the two groups not controlling for other factors, a two-group t test was performed at each visit.

## Results

Of the original 100 patients in the intent-to-treat population, 97 were eligible for the pooled analysis. Three patients were not eligible because they either lacked a recorded date for onset of signs of aHUS or had a baseline eGFR > 90 ml/min/1.73 m^2^. Sixty-eight (70 %) patients had data at 12 months follow-up on eculizumab.

Stratification resulted in 21 patients in the group starting eculizumab at ≤7 days of presentation of TMA, and 76 patients starting at >7 days of presentation. Baseline demographics and disease characteristics are shown in Table 1 (supplementary online Tables 1 and 2 present baseline demographic data and key characteristics, on an individual trial basis). Significant differences could be seen in some baseline parameters between the two groups. Among patients in the ≤7 day group there were more patients <18 years of age, a higher median LDH level, a lower median platelet count at baseline, a smaller proportion receiving PE/PI at baseline and a shorter duration of dialysis compared to the >7 day group. Median eGFR at baseline was similar: 11 (range 6–53) and 16 (7–76) ml/min/1.73 m^2^, in the ≤7 day vs. >7 day groups, respectively (p = 0.30).

**Table 1:** Baseline demographics and disease characteristics of the 97 patients with aHUS included in the pooled analysis

Characteristic	Time to treatment			p*
	≤ 7 days, *n* = 21	>7 days, *n* = 76	All, *n* = 97	
Median age, years (range)	30 (0–69)	29 (0–80)	29 (0–80)	
Age group in years, *n* (%)				
<18	10 (48)	15 (20)	25 (26)	0.029^†^
≥18	11 (52)	61 (80)	72 (74)	
Female gender, *n* (%)	11 (52)	49 (64)	60 (62)	0.323^†^
Complement mutation or autoantibody, *n* (%)				
Any mutation or autoantibody	9 (43)	48 (63)	57 (59)	0.133^†^
CFH mutation	5 (24)	19 (25)	24 (25)	
No complement mutation or autoantibody, *n* (%)	12 (57)	28 (37)	40 (41)	
Median time from last aHUS manifestation to eculizumab treatment, months (range)	0.13 (0.03–0.20)	1.02 (0.23–47.40)	0.75 (0.03–47.40)	–
Median number of TMA events, n (range)	1 (1–6)	1 (1–9)	1 (1–9)	0.421^†^
Receiving PE/PI at baseline, *n* (%)	11 (52)	60 (79)	71 (73)	0.001^†^
Median PE/PI duration during last aHUS manifestation prior to first dose, months (range)	0.10 (0.03–0.20)	0.67 (0.03–46.46)	0.49 (0.03–46.46)	<0.001^§^
Dialysis at baseline, *n* (%)	12 (57)	31 (41)	43 (44)	0.219^†^
Median dialysis duration during last aHUS manifestation prior to first dose, months (range)	0.05 (0.03–0.20)	0.39 (0.03–34.85)	0.30 (0.03–34.85)	0.007^†^
History of kidney transplantation, *n* (%)	7 (33)	19 (25)	26 (27)	0.578^†^
Median baseline platelet count × 10^9^/l (range)	81.5 (18.0–193.0)	133.5 (16.0–420.5)	127.5 (16.0–420.5)	0.002^§^
Platelet count <150 × 10^9^/l, *n* (%)	19 (90)	45 (59)	64 (66)	0.008^†^
Median hemoglobin, mg/dl (range)	n = 18 84.0 (41.0–117.0)	n = 71 92.0 (54.0–131.0)	n = 89 89.0 (41.0–131.0)	0.122^§^
Median LDH, U/l (range)	669.1 (131.0–7164.0)	297.5 (134.0–3682.0)	343.0 (131.0–7164.0)	<0.001^§^
Median creatinine, µmol/l (range)	n = 20 214.0 (112.0–1007.8)	n = 74 243.1 (28.0–1169.6)	n = 94 238.7 (28.0–1169.6)	0.708^§^
Median baseline eGFR, ml/min/1.73 m^2^ (range)^a^	11.0 (5.6–53.2)	16.0 (7.3–76.1)	15.9 (5.6–76.1)	0.299^§^

*aHUS* atypical hemolytic uremic syndrome, *CFH* complement factor H, *eGFR* estimated glomerular filtration rate, *LDH* lactate dehydrogenase, *PE/PI* plasma exchange/plasma infusion, *TMA* thrombotic microangiopathy

* Comparison between ≤7 and >7 day groups

^†^p values calculated using the Fisher exact test

^§^p values calculated using the Wilcoxon rank-sum test

^a^eGFR for patients on dialysis was imputed to 10 ml/min/1.73 m^2^

The final model obtained through stepwise forward variable selection contained significant main effects: time to treatment, age group, baseline LDH, and hemoglobin. Significant interaction terms that remained in the final model were visit (post-dose visits in months) by time to treatment, visit by age group, visit by baseline LDH, visit by baseline hemoglobin, and age group by baseline hemoglobin (Table 2). The final repeated measures analysis model identified above was rerun, replacing the continuous variable of time to treatment with the categorical time to treatment (≤7 and >7 days). Results of these models are presented in Table 2.

**Table 2:** Repeated measures analysis of eGFR change from baseline to post-treatment through 12 months

Effect^a^	Time to treatment as continuous variable		Time to treatment as categorical variable (≤7 vs. >7 days)	
	Coefficient	p	Coefficient	p
Time to treatment (days)	−0.03	0.0181	−2.8	0.3569
Age group (adult vs. child)	−84.8	0.0061	−88.5	0.0053
Baseline LDH (U/l)	0.01	0.0078	0.01	0.0016
Baseline hemoglobin (g/l)	−0.97	0.0002	−1.19	<0.0001
Trial visit	–	<0.0001	–	<0.0001
Baseline eGFR	0.21	0.1964	0.12	0.4539

*eGFR* estimated glomerular filtration rate, *LDH* lactate dehydrogenase

^a^Interaction terms that remain significant in the final model are visit (scheduled post-dose visits in months) by time to treatment, visit by age group, visit by baseline LDH, visit by baseline hemoglobin, and age group by baseline hemoglobin

The final multivariate analysis model showed that time to treatment as a continuous variable was a significant factor in determining eGFR change from baseline when controlling for other factors such as age group, baseline LDH, hemoglobin, eGFR and post-dose visits. Time to initiation of eculizumab treatment when dichotomized into groups for ≤7 and >7 days was not significant when controlling for these other factors.

**Fig. 1:**
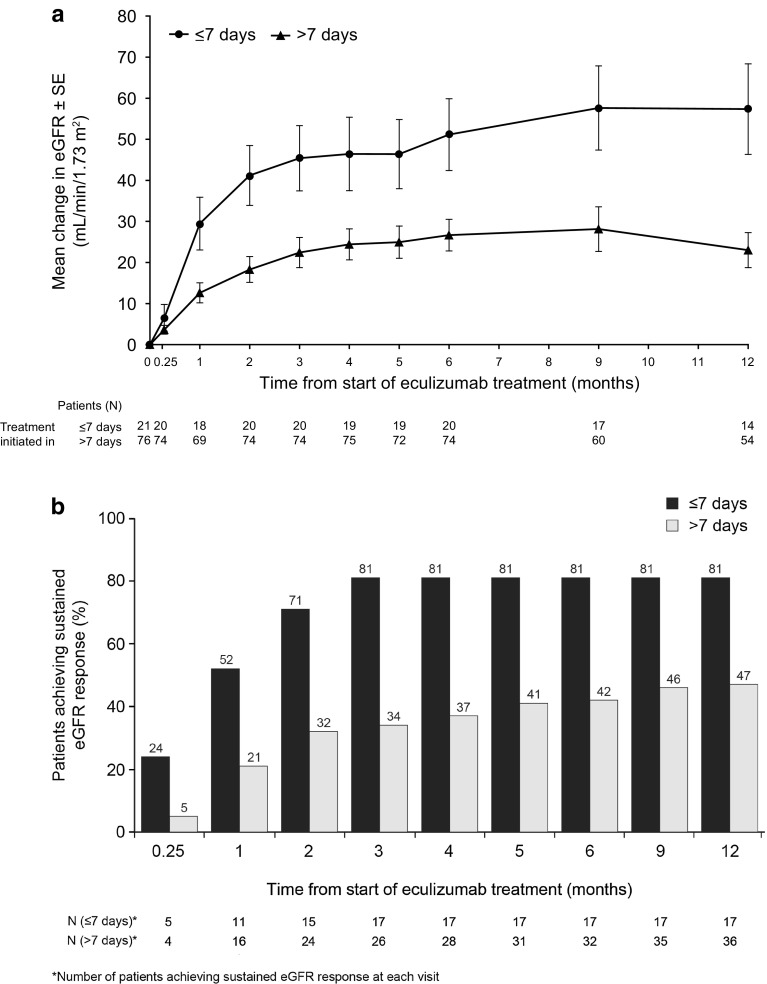
Improvements in eGFR over time. **a** Raw mean change in eGFR among patients receiving eculizumab ≤7 or >7 days after onset of last aHUS manifestation, and **b** Proportion of patients achieving sustained response of an increase in eGFR of ≥15 ml/min/1.73 m^2^. *aHUS* atypical hemolytic uremic syndrome, *eGFR* estimated glomerular filtration rate, *SE* standard error

When eGFR change from baseline at each visit was tested separately between the two groups using a two-group t-test, patients receiving eculizumab ≤7 days after first signs of the last aHUS manifestation showed a significantly (p < 0.05) greater mean improvement in eGFR from month 1 onwards. At 1 year, the eGFR increase from baseline was 57 ml/min/1.73 m^2^ in the ≤7 day group compared with 23 ml/min/1.73 m^2^ in the >7 day group (Fig. 1a).

**Fig. 2:**
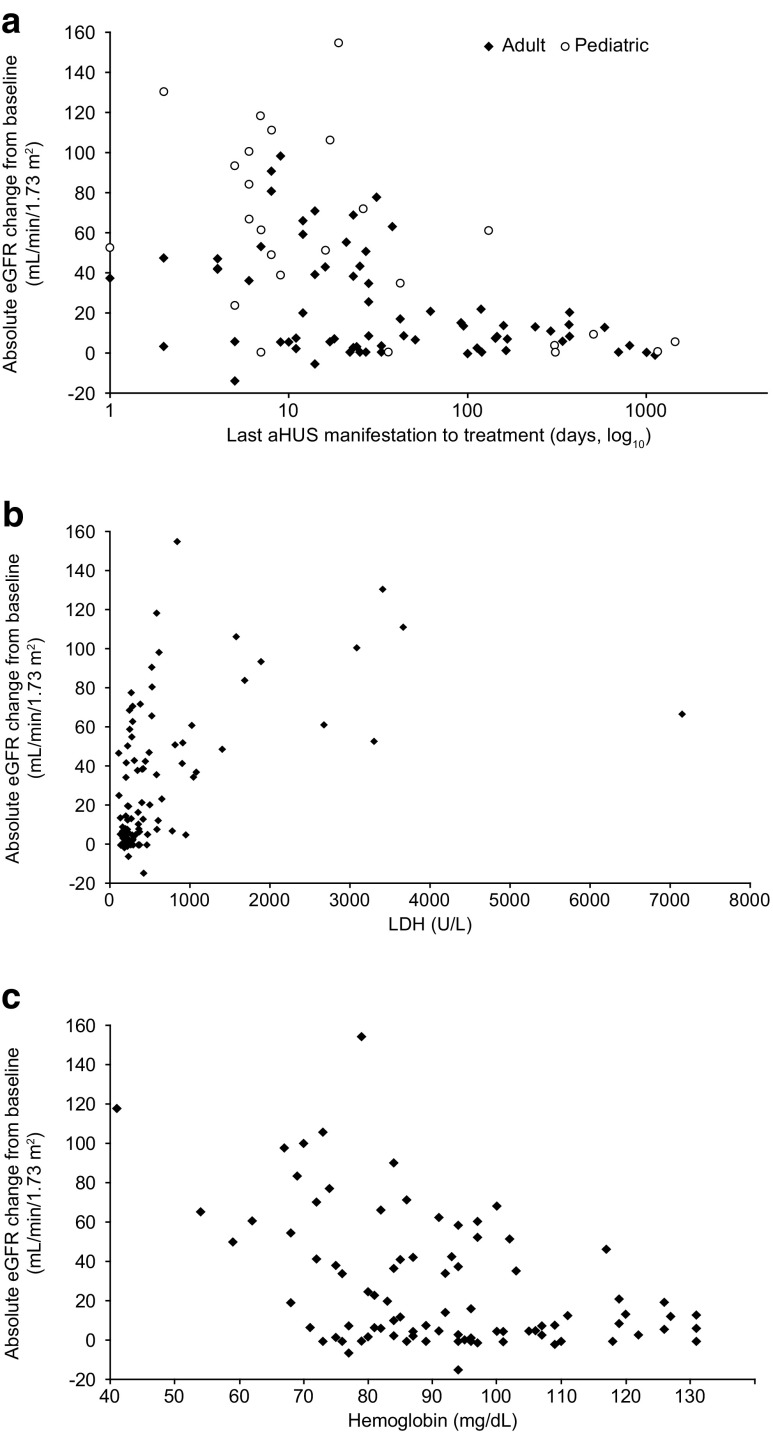
Scatter plots showing change from baseline in eGFR at 6 months for individual patients for each parameter according to baseline value. **a** Time from last aHUS manifestation to eculizumab treatment by age group; **b** LDH and **c** hemoglobin. The time to initiation of treatment is presented on a log-scale axis for clarity. *aHUS* atypical hemolytic uremic syndrome, *eGFR* estimated glomerular filtration rate, *LDH* lactate dehydrogenase

Time to treatment and age group both remained in the final model as significant factors. A scatter plot of eGFR change from baseline to 6 months versus time to treatment showed a similar trend in the relationship between eGFR change and time to treatment in both children and adults (Fig. 2a). LDH and hemoglobin were also significant predictors of eGFR change from baseline, indicating that patients with higher LDH and/or lower hemoglobin are expected to have a greater eGFR increase after treatment (Fig. 2b, c).

Seventeen patients (81 %) in the ≤7 day group had achieved a sustained eGFR response (≥15 ml/min/1.73 m^2^ for at least 28 days) at 3 months follow-up. This proportion remained stable through 1 year. In contrast, 26 (34 %) and 36 (47 %) patients in the >7 days group achieved a sustained eGFR at 3 months and at 1 year, respectively (Fig. 1b). The proportion achieving a sustained eGFR response (≥15 ml/min/1.73 m^2^) was significantly higher (p < 0.05) for the ≤7 day group compared to the >7 day group at all visits based on Fisher’s exact test.

Of the pooled cohort of 97 patients, 64 had an abnormal platelet count at baseline. Platelet counts normalized in 60 of these patients, 18 (86 %) in the ≤7 day group and 42 (55 %) in the >7 day group. Median time to platelet count normalization was similar in the two groups (7 vs. 7.5 days), although the range of values in the ≤7 day (1–80 days) group was narrower than in the >7 days group (1–189 days). Corresponding mean (SD) times to platelet normalization were 14.3 ± 20.3 and 27.8 ± 38.7 days, respectively (p = 0.083).

## Discussion

aHUS is a severe disease with poor outcomes in which progression can be rapid; prior to the availability of eculizumab over 50 % of patients with aHUS died, required dialysis or developed permanent kidney damage in the first year after diagnosis [3]. This indicates a need for urgent treatment of the underlying disease to prevent continuing organ damage. Observational data show improved recovery of renal function (decreased serum creatinine levels) in five patients who received eculizumab therapy within 28 days of the start of the last aHUS event when compared to 7 patients who received treatment after ≥28 days [9]. A significant correlation was also found between time to initiation of eculizumab treatment after the last presentation and graft function recovery in 8 patients with kidney transplantation [9].

This post hoc multivariate analysis of the time from last aHUS manifestation to treatment suggests that a shorter time to treatment with eculizumab results in a significantly better renal outcome for patients with aHUS. This is the largest group of patients with aHUS in whom the clinical impact of the timing of eculizumab initiation relative to presentation with disease has been explored. The analysis presents data up to 1-year because this was considered sufficient follow-up to detect differences in outcomes without excessive loss of patients available for evaluation over time. Renal outcomes were significantly better in the group of patients initiating eculizumab treatment in ≤7 days of presentation of TMA than in patients initiating treatment after 7 days. Of interest, the sustained improvement of >15 ml/min/1.73 m^2^ was achieved more rapidly in the ≤7 day group while improvement progressed more slowly in the >7 day group, indicating the possibility to also continue eGFR improvement after 6 months of eculizumab treatment.

Factors other than early treatment initiation with eculizumab that were independently associated with eGFR outcome were younger age, higher LDH, and lower hemoglobin. Clinically it can be hypothesized that early treatment and younger age are suggestive of less renal damage at treatment initiation and, therefore, possibly greater potential of recovery of renal function.

Overall, these data support the importance of prompt treatment of aHUS to optimize recovery of renal function and to avoid dialysis or transplantation in both adults and pediatric patients, as reported elsewhere [3, 12, 13]. The relationship between AKI and CKD is well established [6, 14–17], and as such the avoidance or minimization of AKI is important for the long-term maintenance of kidney function without the need for dialysis or transplantation, including in patients with aHUS [6].

Recent guidelines recommend treating children and adults with a diagnosis of aHUS with eculizumab within 24 h. In patients where further investigations are required, and where no significant improvement of both renal and hematological parameters is observed, eculizumab should be initiated after no more than 5 plasma exchanges when aHUS diagnosis has been confirmed [9, 12]. The findings of this study further support these guidelines.

No significant difference in time to platelet count normalization was shown between patients receiving treatment in ≤7 or >7 days, although a trend favoring earlier treatment was evident from mean numbers of days to normalization and ranges of times around median values. In the original source studies, platelet counts normalized rapidly: for example, in the two published trials which enrolled patients aged ≥12 years, 53 % of those with abnormal platelet counts at baseline had normal counts by day 7 [10]. Of note in this context, as illustrated in a case previously reported by Dorresteijn et al., platelet counts are not always a reliable marker of improvement in aHUS [18], and TMA may still progress in patients with normal platelet counts [19].

Limitations of this study include the non-randomized study design and the retrospective nature of the analysis, although the source data were collected prospectively. Further, it is impossible to know how patients treated in ≤7 days would have responded if treatment had been delayed until >7 days. Also, patients who may have already been receiving dialysis for an extended period before starting eculizumab therapy may not have been expected to show major improvements in eGFR, which might have resulted in bias favoring the ≤7 day group. The median time on dialysis was, however, fairly short (12 days) in the >7 day group with only two patients on dialysis for >90 days. It is also not possible to exclude a tendency for the initiation of eculizumab more rapidly in patients presenting with more severe symptoms.

In summary, the results of this large pooled analysis indicate overall that patients with aHUS derive greater and more sustained eGFR recovery benefit from treatment with eculizumab when therapy is started earlier. Our data concur with previous observations and treatment guidelines that recommend prompt treatment with eculizumab in patients diagnosed with aHUS.

## Electronic supplementary material

Below is the link to the electronic supplementary material.

Supplementary material 1 (DOCX 15 kb)

Supplementary material 2 (DOCX 14 kb)
