# Supplementary material for: Improved renal recovery in patients with atypical hemolytic uremic syndrome following rapid initiation of eculizumab treatment
Source: J Nephrol. 2016 Mar 19;30(1):127–34. doi: 10.1007/s40620-016-0288-3 (PMC5316393; doi:10.1007/s40620-016-0288-3)
Supplement: Supplementary file 1 — Supplementary material 1 (DOCX 15 kb) [file 40620_2016_288_MOESM1_ESM.docx]

**Supplementary online Table 1** Baseline demographics and disease characteristics of the 97 patients with aHUS included in the pooled analysis according to source trial

| Characteristic | C08-002 A/B (n=17) | C08-003 A/B  (n=20) | C10-003  (n=19) | C10-004  (n=41) |
| --- | --- | --- | --- | --- |
| Median age, years (range) | 28 (17–68) | 27.5 (13–63) | 6 (0–17) | 35 (18–80) |
| Age group in years, n (%)  <18  ≥18 | 1 (6)  16 (94) | 5 (25)  15 (75) | 19 (100)  0 (0) | 0 (0)  41 (100) |
| Female gender, n (%) | 12 (71) | 12 (60) | 8 (42) | 28 (68) |
| Complement mutation or autoantibody, n (%)  Any mutation or autoantibody  CFH mutation  No complement mutation or autoantibody, n (%) | 13 (76)  6 (35)  4 (24) | 14 (70)  5 (25)  6 (30) | 9 (47)  2 (11)  10 (53) | 21 (51)  11 (27)  20 (49) |
| Median time from last aHUS manifestation to eculizumab treatment, months (range) | 0.80  (0.20–3.70) | 10.60  (3.00–47.40) | 0.20  (0.03–4.26) | 0.52  (0.03–19.15) |
| Median number of TMA events, n (range) | 2 (1–5) | 3.5 (1–9) | 1 (1–2) | 1 (1–6) |
| Receiving PE/PI at baseline, n (%) | 17 (100) | 20 (100) | 8 (42) | 32 (78) |
| Median PE/PI duration during last manifestation prior to first dose, months (range) | 0.15  (0.03–1.05) | 3.91  (0.03–46.61) | 0.23  (0.03–0.69) | 0.33  (0.03–3.68) |
| Dialysis at baseline, n (%) | 6 (35) | 2 (10) | 11 (58) | 24 (59) |
| Median dialysis duration during last manifestation prior to first dose, months (range) | 0.30  (0.03–0.59) | 0.46  (0.07–34.99) | 0.20  (0.03–1.15) | 0.33  (0.03–0.82) |
| History of kidney transplantation, n (%) | 7 (41) | 8 (40) | 2 (11) | 9 (22) |
| Median baseline platelet count  x 10^9^/L (range) | 117.5  (62.0–160.5) | 218.3  (104.5–420.5) | 107.0  (20.0–146.0) | 125.0  (16.0–332.0) |
| Platelet count <150 x 10^9^/L, n (%) | 15 (88) | 3 (15) | 19 (100) | 27 (66) |
| Median hemoglobin, mg/dL (range) | 87  (67–126) | 108  (79–131) | 80  (41–102) | 87  (54–131) |
| Median LDH, U/L (range) | 269  (134–634) | 200  (151–391) | 1064  (282–7164) | 375  (131–3318) |
| Median creatinine, µmol/L (range) | 256.4  (123.7–786.8) | 234.3  (106.1–892.9) | 131.0  (28.0–449.0) | 349.2  (112.0–1169.6) |
| Median baseline eGFR, mL/min/1.73m^2^ (range)^#^ | 19.1  (10.0–58.8) | 28.4  (7.6–72.3) | 10.0  (10.0–76.1) | 10.0  (5.6–52.5) |
| ^#^eGFR for patients on dialysis was imputed to 10 mL/min/1.73m^2^  *aHUS* atypical hemolytic uremic syndrome, *CFH* complement factor H*, eGFR* estimated glomerular filtration rate, *LDH* lactate dehydrogenase, *PE/PI* plasma exchange/plasma infusion, *TMA* thrombotic microangiopathy | | | | |
