# Supplementary material for: Improved renal recovery in patients with atypical hemolytic uremic syndrome following rapid initiation of eculizumab treatment
Source: J Nephrol. 2016 Mar 19;30(1):127–34. doi: 10.1007/s40620-016-0288-3 (PMC5316393; doi:10.1007/s40620-016-0288-3)
Supplement: Supplementary file 2 — Supplementary material 2 (DOCX 14 kb) [file 40620_2016_288_MOESM2_ESM.docx]

**Supplementary online Table 2.** Key characteristics of the four clinical trials included in the pooled analysis

|  | C08-002 A/B (n=17) | C08-003 A/B  (n=20) | C10-003  (n=22) | C10-004  (n=41) |
| --- | --- | --- | --- | --- |
| Design | Prospective, single arm, open label | | | |
| Trial registration number | NCT00844545, NCT00844844 | NCT00838513, NCT00844428 | NCT01193348 | NCT01194973 |
| Key eligibility | Adults and adolescents (≥12 years of age) | Adults and adolescents (≥12 years of age) | Pediatric patients (1 month to 18 years of age) | Adults (>18 years of age) |
|  | Signs of hemolysis,  thrombocytopenia, and  renal impairment | Signs of hemolysis, and renal impairment | Signs of hemolysis,  thrombocytopenia, and  renal impairment | Signs of hemolysis,  thrombocytopenia, and  renal impairment |
|  | ≥4 PE/PIs 1 week prior  to screening | ≥1 PE/PI every 2 weeks;  no more than 3 per week,  frequency unchanged for  8 weeks | PE/PI <5 weeks | No PE/PI requirement |
|  | No chronic dialysis | Chronic dialysis allowed | No chronic dialysis | No chronic dialysis |
|  | ADAMTS13 >5 % | ADAMTS13 >5 % | ADAMTS13 >5 % | ADAMTS13 >5 % |
|  | No evidence of STEC-HUS or Shiga toxin | No evidence of STEC-HUS or Shiga toxin | No evidence of STEC-HUS or Shiga toxin | No evidence of STEC-HUS or Shiga toxin |
| Primary endpoint | Platelet count change  from baseline to 26-weeks of treatment | TMA event-free status* | Complete TMA response^†^ | Complete TMA response^§^  Submission complete TMA response^†^ |
|  | Complete TMA response^†^ | Complete TMA response^†^ |  |  |
| *Absence of a decrease in platelet count by >25 % for ≥12 weeks, PE/PI, or new dialysis; ^†^ Normalization of platelet count and lactate dehydrogenase level, and ≥25 % decrease in serum creatinine level from baseline; ^§^Normalization of platelet count and lactate dehydrogenase level, and preservation of renal function (no increase in serum creatinine level from baseline)  *ADAMTS13* a disintegrin and metalloproteinase with a thrombospondin type 1 motif member 13, *PE/PI* plasma exchange/plasma infusion, *STEC-HUS* Shiga-like toxin-producing *E. coli*, *TMA* thrombotic microangiopathy | | | | |
